# Mortality and causes of death among long-term care residents in Helsinki, Finland 2017–2021, a longitudinal cohort study

**DOI:** 10.1177/14034948251331314

**Published:** 2025-05-01

**Authors:** Lara Kuokkanen-Estrada, Hanna-Maria Roitto, Ulla L Aalto, Satu K. Jyväkorpi, Riitta K.T. Saarela, Hannu Kautiainen, Kaisu H Pitkälä

**Affiliations:** 1 Clinicum, Faculty of Medicine, University of Helsinki, Finland; 2 Clinics of Internal Medicine and Rehabilitation, Department of Geriatrics, University of Helsinki and Helsinki University Hospital, Finland; 3 Department of General Practice and Primary Health Care, University of Helsinki, Finland; 4 National Nutrition Council, Finland; 5 Social Services, Health Care and Rescue Services Division, Oral Health Care, City of Helsinki, Finland

**Keywords:** Long-term care, mortality, dementia, cause of death

## Abstract

**Aims:**

Knowledge about mortality and causes of death is needed to improve the quality of end-of-life care in long-term care (LTC). This study aimed to investigate mortality and causes of deaths in LTC. We explored how age, sex, and having dementia at baseline were associated with the standardized mortality ratio (SMR) and causes of death.

**Methods:**

Participants comprised residents (*N* = 2513) living in LTC facilities in Helsinki, Finland in March 2017. Background data on demographics, diagnoses, and medications were retrieved from medical charts. Causes of death were retrieved from the Finnish Cause of Death Register for deaths that were registered up to 31 December 2021.

**Results:**

Of the 2513 participants, 2033 died during follow-up. All-cause SMR was 3.31 (95% CI 3.17–3.46) compared with the same-aged general population in Finland. SMR for men was 4.33 (95% CI 3.98–4.70), for women was 3.05 (95% CI 2.90–3.20), and for residents younger than 70 years 13.80 (95% CI 11.17–17.05). SMR decreased linearly with age. There was no difference in SMR among those with and without dementia. For both sexes the most common cause of death was dementia (67.0%), followed by cardiovascular disease (CVD; 15.9%), neurological diseases (5.2%), and cancer (3.1%). The most common cause of death in residents with dementia was dementia (77.7%), whereas in residents without dementia it was CVD (36.0%).

**Conclusions:**

**Male sex and younger age were associated with a higher SMR. Residents most frequently died of dementia. End-of-life care in LTC needs to be tailored to meet the needs of people with dementia.**

## Background

Long-term care (LTC) residents are living the last years of their life. According to previous studies the median survival in LTC is a little over 2 years [1-3]. Certain comorbidities such as hypertension, atrial fibrillation, and higher functional and cognitive impairment have been shown to increase the risk of mortality [4]. Higher mortality among nursing home residents has also been associated with underweight [1, 5], older age [1-3], a higher prevalence of comorbidities [1, 2], greater dependency in activities of daily living [1, 3], male gender, and hospitalizations prior to nursing home admission [2, 3]. Thus, the focus has been more on mortality-related factors than on how mortality rates in LTC populations compare with age-specific mortality rates in the general population, that is, standardized mortality ratios (SMRs).

Dementia is one of the main risk factors for the institutionalization of older adults [6]. People often live for years with dementia, which is a progressive and incurable condition. At the beginning of the cognitive decline trajectory it can be difficult to think of dementia as a terminal condition [7]. While dementia is the seventh leading cause of death worldwide [8], among the oldest seniors, dementia is rapidly increasing as a cause of death and LTC facilities as places of death [9]. LTC residents with dementia are often not perceived as having a terminal condition, and therefore may not receive optimal palliative care [10]. On the other hand, most older adults with dementia have multimorbidity and not all people with dementia die of the condition [11, 12].

Even though numerous studies have been conducted on mortality risk factors and on causes of death in LTC, discrepancies and heterogeneity in cause-of-death coding pose challenges in international comparisons [13]. One challenge is that dementia and causes leading to dementia, like Alzheimer’s disease, can fall under various cause-of-death categories including psychiatric disorder and neurological disease and therefore might not be seen as being on the same continuum. Some previous studies report immediate cause of death whereas World Health Organization (WHO) guidelines recommend using the underlying cause of death for mortality statistics [14]. For example, an Italian study from 2020 reported the most common causes of death in recently admitted LTC residents to be circulatory system diseases (39.8%) followed by dementia (13.3%). Separately, 3.4% were reported to have died from Alzheimer’s disease [15]. In a Norwegian study from 2022, dementia was the most common underlying cause of death (37.7%), including both dementia and Alzheimer’s disease diagnoses, followed by cardiovascular disease (CVD) (28.8%) [16]. In this same Norwegian study, the percentage of dementia rose to 62.3% when both underlying and immediate causes of death were taken into account. Thus, the findings have been highly inconsistent between studies.

## Aims

The aim of this study was to add to current knowledge to improve the quality of end-of-life care, by investigating mortality and causes of death in LTC facilities in Helsinki, Finland. In addition, we investigated how age and sex were associated with SMRs and compared causes of death by sex and in residents with and without dementia.

## Methods

### Participants

The participants consisted of residents (*N* = 3895) living in all LTC facilities (including nursing homes and assisted living facilities) in Helsinki in 2017. Exclusion criteria were patient refusal or having moderate to severe dementia (Clinical Dementia Rating (CDR) 2–3) without any proxy available to give informed consent. After exclusions, 2513 (64.5%) participants remained. Trained registered nurses performed the assessments and interviews according to a structured study protocol. Baseline data were collected in March 2017, and each resident was assessed over the course of 1 day. In each LTC unit, background data on demographics, education level, and medical diagnoses (acute illnesses and chronic conditions) were retrieved from medical charts. Those who had a dementia diagnosis in their medical chart were counted as having dementia (International Classification of Disease 10th Revision (ICD-10) F00–F03 and G30–G32). The Charlson Comorbidity Index [17] was calculated to assess each resident’s burden of comorbidity.

Medication use was retrieved from medical charts on the assessment day. The mean number of medications used regularly was calculated. The severity of dementia was assessed using the CDR “memory” item (0–0.5 = no or possible cognitive decline, 1 = mild cognitive decline, 2 = moderate cognitive decline, 3 = severe cognitive decline), which is a validated method to assess dementia stage. Dependence in activities of daily living was assessed by the CDR “personal care” item (0–0.5 = fully capable of self-care; 1 = needs occasional prompting; 2 = requires assistance in dressing, personal hygiene, and the keeping of personal belongings; 3 = requires much help with personal care, often incontinent) [18].

The causes of death were retrieved from death certificates from the Finnish Cause of Death Register for deaths that were registered by 31 December 2021 at the latest—we used the underlying cause of death in the study. Eight categories were used for cause of death, coded according to the ICD-10 [14]. The dementia category included codes F00–F03 and G30–G32; the CVD category included codes I00–I99; other neurological disease category codes were G00–G26 and G35–G99, except codes G40.51, G62.1, G72.1 as they were classified as alcohol-related diseases. The cancer category included the codes C00–D48; the external causes of death category codes were V01–X59 and Y85–Y87; the infectious disease codes included A00–B99, J09–J18, J65, U07.1, and U07.2; and the alcohol-related disorder category codes comprised F10, G40.51, G62.1, G72.1, K29.2, K70, K86.0, and K85.2. All other causes of death were classified in the category “Other.”

### Statistics

Characteristics of the participants were expressed as means with standard deviations (SDs), or as counts with percentages. Statistical comparisons between the sexes were performed with the *t*-test and Pearson’s chi-square test. The ratio of observed to expected number of deaths, the SMR for all-cause and specific cause of death, was calculated using the subject-years method with 95% confidence intervals (CIs). The expected number of deaths was determined by multiplying the number of person-years of observation by the appropriate mortality rate in the general population according to the categories sex, 1-year age group, and calendar period in the Finnish population (Official Statistics of Finland). SMRs were used to quantify the risk of death in this cohort compared with the general population. We used a multivariate Cox proportional hazards model to calculate the adjusted hazard ratios (HRs) with 95% CI. All statistical analyses were carried out with Stata, version 18.0 (StataCorp, College Station, TX).

### Ethics

All procedures were performed in accordance with the ethical standards laid down in the 1964 Declaration of Helsinki and its later amendments. The study was approved by the Ethics Committee of the Department of Medicine at Helsinki University Hospital and City of Helsinki (HUS/2042/2016). Informed written consent was obtained from all participants or in the case of moderate to severe dementia (CDR 2–3) from their closest proxies.

## Results

From the whole cohort, a total of 6229 person-years were followed up, 1576 in men and 4653 in women. Of the 2513 participants, 73.4% were women (Table I). The women were older: mean age at baseline for women was 85 years and for men was 80 years. Some 167 participants were under 70 years of age. Men had diabetes, Parkinson’s disease, and stroke more often than women, while women had dementia more than men. No difference emerged in cardiovascular morbidity, cancer, or chronic obstructive pulmonary disease at baseline. The mean number of medications used was 8.7 for women and 9.0 for men. According to the CDR classification women had poorer cognition than men (*p* < 0.001), but there was no difference in dependence for personal care (*p* = 0.053).

**Table I.** Characteristics of residents grouped by sex.

	Women*N* = 1844	Men *N* = 669	*P*-value
Age, mean (SD)	84.7 (8.2)	79.5 (9.0)	<0.001
Education <8 years, *n* (%)	726 (39.4)	221 (33.0)	0.003
Charlson Comorbidity Index, mean (SD)	2.2 (1.4)	2.3 (1.5)	0.027
Medical history of . . ., *n* (%) Diabetes Ischemic heart disease Atrial fibrillation Stroke Dementia Parkinson’s disease COPD Cancer	329 (17.8) 355 (19.3) 318 (17.2) 391 (21.2) 1466 (79.5) 85 (4.6) 168 (9.1) 204 (11.1)	163 (24.4) 118 (17.6) 109 (16.3) 201 (30.0) 467 (69.8) 56 (8.4) 72 (10.8) 72 (10.8)	0.002 0.36 0.57 <0.001 <0.001 <0.001 0.21 0.83
Number of medications, mean (SD)	8.7 (3.6)	9.0 (3.6)	0.12
Mild to severe dementia, (CDR memory class 1–3), *n* (%)	1507 (81.7)	496 (74.1)	<0.001
Dependent for personal care, (CDR personal care class 2–3), *n* (%)	1657 (89.9)	583 (87.1)	0.053
Deceased, *n* (%)	1483 (80.4)	550 (82.2)	0.31

*Note*. Charlson Comorbidity Index [17]; COPD: chronic obstructive pulmonary disease; CDR: Clinical Dementia Rating [18].

**Figure 1.**
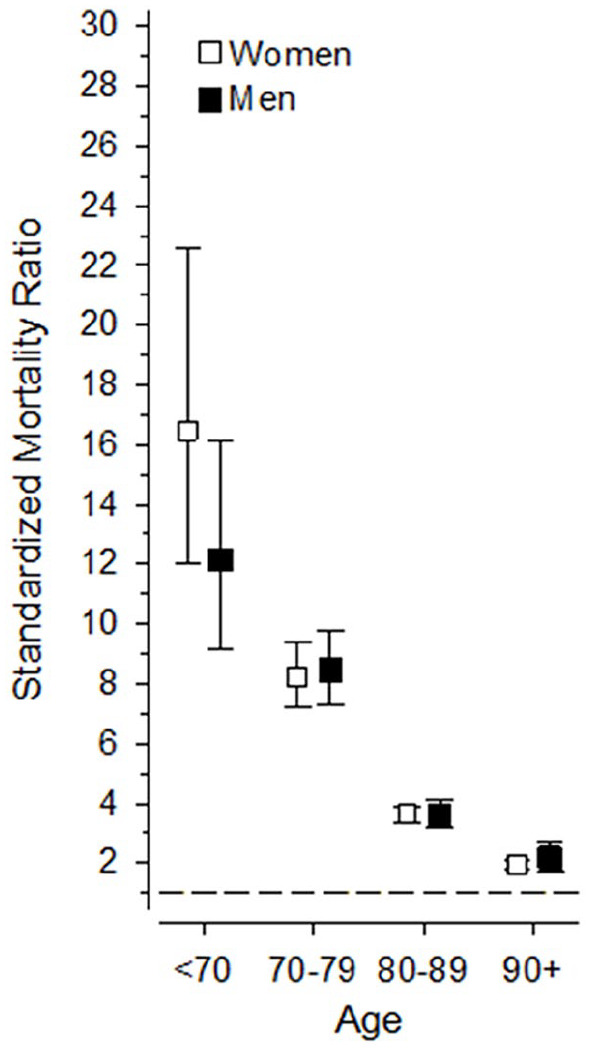
Standardized mortality ratio according to age and sex.

Of all residents, 2033 (80.9%) (1483 women (80.4%) and 550 men (82.2%)) died during follow-up. The SMR for the whole sample was 3.31 (95% CI 3.17–3.46). The SMR for women was 3.05 (95% CI 2.90–3.20) and for men was 4.33 (95% CI 3.98–4.70). The SMR decreased linearly with age (*p* < 0.001) (Figure 1). Younger residents in both sexes had a significantly higher SMR than older residents. Having dementia at baseline did not affect all-cause SMR. Residents with dementia at baseline had an SMR of 3.29 (95% CI 3.13–3.45) and those without dementia 3.42 (95% CI 3.11–3.76). All residents having dementia as a cause of death had an SMR of 7.00 (95% CI 6.64–7.38). The SMR for women was 6.30 (95% CI 5.93–6.69) and 11.40 (95% CI 10.19–12.76) for men.

The most common cause of death in all residents was dementia (67.0%), followed by CVD (15.9%) and then other neurological diseases (5.2%), and cancer (3.1%). In younger residents (<70 years), dementia was still the predominant cause of death (39.5%), but other causes were more common than in older residents: CVD (15.1%), other neurological diseases (12.8%), and alcohol-related disorders (11.6%). The underlying cause of death was an infection for 30 residents. A total of 19 residents died from COVID-19 infection: 14 occurred in 2020 and 5 in 2021.

Dementia was the most common cause of death for both women and men, but there were differences in the prevalence of causes of death between the sexes (Table II). As a cause of death, the proportion of dementia was higher for women (71.3%) than for men (55.3%). All the other cause-of-death categories were more common among men. There was a clear difference in alcohol-related deaths, as 0.5% of women and 4.0% of men died from alcohol-related diseases.

**Table II.** Causes of death grouped by sex.

Cause of death	All, *N* = 2033, *n* (%)	Women, *N* = 1483 *n* (%)	Men, *N* = 550 *n* (%)	*P*-value between sex
Dementia	1362 (67.0)	1058 (71.3)	304 (55.3)	<0.001
Cardiovascular disease	323 (15.9)	218 (14.7)	105 (19.1)	0.012
Other neurological diseases	105 (5.2)	65 (4.4)	40 (7.3)	0.007
Cancer	63 (3.1)	39 (2.6)	24 (4.4)	0.037
External causes of death	37 (1.8)	21 (1.4)	16 (2.9)	0.021
Infectious disease	30 (1.5)	18 (1.2)	12 (2.2)	0.095
Alcohol-related disorders	29 (1.4)	7 (0.5)	22 (4.0)	<0.001
Other	84 (4.1)	57 (3.8)	27 (4.9)	0.29

Of those who had dementia at baseline, 77.7% died of dementia (Table III). The main competing cause of death in residents with dementia was CVD (10.6%), whereas other causes were less prevalent. In residents without dementia at baseline the most common cause of death was CVD (36.0%), followed by dementia (26.6%), and other neurological diseases (11.5%).

**Table III.** Causes of death grouped by dementia status.

Cause of death	All, *N* = 2033 *n* (%)	Dementia at baseline, *N* = 1608 *n* (%)	No dementia at baseline, *N* = 425 *n* (%)	*P*-value between those with and without dementia *n* (%)
Dementia	1362 (67.0)	1249 (77.7)	113 (26.6)	<0.001
Cardiovascular disease	323 (15.9)	170 (10.6)	153 (36.0)	<0.001
Other neurological diseases	105 (5.2)	56 (3.5)	49 (11.5)	<0.001
Cancer	63 (3.1)	34 (2.1)	29 (6.8)	<0.001
External causes of death	37 (1.8)	25 (1.6)	12 (2.8)	0.17
Infectious disease	30 (1.5)	19 (1.2)	11 (2.6)	0.076
Alcohol-related disorders	29 (1.4)	23 (1.4)	8 (1.9)	0.72
Other	84 (4.1)	32 (2.0)	50 (11.8)	<0.001

A multivariate Cox proportional hazard model for dementia mortality showed a higher risk of mortality for men (*p* = 0.015), older residents (*p* < 0.001) and for those dependent for personal care (*p* < 0.001) (Table IV).

**Table IV.** Multivariate Cox proportional hazard model for dementia mortality.

Variables	HR (95% CI)	*P*-value
Male sex	1.19 (1.04–1.38)	0.015
Mean age	1.07 (1.06–1.08)	<0.001
Education <8 years	0.99 (0.91–1.08)	0.83
Charlson Comorbidity Index	1.02 (0.98–1.06)	0.35
Dependent for personal care, (CDR personal care class 2–3)	2.23 (1.77–2.81)	<0.001

*Note*. HR: hazard ratio; CDR: Clinical Dementia Rating.

## Discussion

According to our results over 80% of the residents died during the 4.5-year follow-up. The most common cause of death in all residents was dementia, followed by CVD. Mortality in the whole sample was significantly higher compared with the same-aged general population, especially for men and younger residents. Of those with dementia at baseline, almost 80% died of dementia, whereas those without dementia at baseline most frequently died of CVD.

The SMR in our study is in line with earlier published data. In previous studies from the UK, the mortality risk for nursing home residents was estimated to be three and four times the expected mortality of their community counterparts [19, 20]. During recent decades, public policy in Finland has been to reduce institutional care for older people. Thus, only those who have severe dementia, mobility disability, or other severe health complications are offered a place in LTC, either in a nursing home or in an assisted living facility, which explains the higher mortality in LTC facilities compared to community-dwelling older adults.

In our study, the SMR was higher for men and for younger residents of both sexes. The higher SMR for residents aged under 70 in our study could be explained by the fact that people under 70 years of age living at home are in better health compared to those in LTC. There was no difference between the all-cause SMR for residents with or without dementia at baseline since all those residing in LTC are approaching end of life, but for different reasons and with different illnesses. The dementia-related SMR was notably higher, indicating a higher dementia-related mortality risk in LTC residents than in their community-dwelling peers with dementia, probably explained by differences in the stage of dementia severity. There was a gender difference in the SMR for residents having dementia as a cause of death. This could be partly explained by the fact that the men were younger, as younger residents had a higher SMR. Men also had Parkinson’s disease and stroke at baseline more frequently than women. Thus, a mixed pathology may have been a more prevalent underlying cause of dementia among the male residents. Older age, male sex, and higher dependency for personal care at baseline predicted earlier mortality.

The results of our study regarding the causes of death are only partly in line with previous findings. Our results differed significantly from a study conducted in Italy, in which the leading causes of death were CVD s [15]. In this Italian study the residents were newly admitted to LTC, which may explain the differences. The authors stated that the first months after admission represented a higher risk of mortality, especially for recently hospitalized patients. Thus, the population studied might differ from ours. In our study the proportion of dementia as the underlying cause of death was significantly higher (67.0%) than in the LTC population in Norway (37.7%) [16], England (31.2%) [21], and Italy (13.3%) [15]. This could be partly explained by coding, as we grouped both dementia and Alzheimer’s disease in the dementia category, something that was not done in the Italian study for example. In Finland, cognition is always tested before permanent placement in an LTC facility. This approach may partly explain the high proportion of dementia at baseline (76.9%) as well as the high proportion of dementia as an underlying cause of death. The results from the Palliative Care for Older People (PACE) study are in accord with our findings. In the PACE study, which described the clinical characteristics of LTC residents in six European countries, the proportion of dementia at time of death was highest in Finland (82.5%), followed by the other countries, ranging from 60.2% (UK) to 77.0% (Italy) [22].

The proportion of dementia as a cause of death in residents with dementia (77.7%) was also high when compared to those living with dementia in the community. In a Finnish study published in 2020, 51% of community-dwellers with clinically verified Alzheimer’s disease had any form of dementia listed as an underlying, direct or intervening cause of death [12]. This is in line with the fact that that severe dementia is one of the main risk factors for institutionalization and therefore the role of dementia as a cause of death in LTC residents is higher than among community-dwelling older adults.

The proportion of infectious diseases, 1.5%, was low in our study compared with international literature. Vossius et al. [16] reported infections accounted for 7.7% of the underlying causes of death. In an Italian nursing home, the proportion of infectious diseases was 4.2% and pneumonia, which was counted separately, was a cause of death in 4.0% of residents [15]. Similarly, in 2014, 4.2% of care home residents in the UK had pneumonia and flu as their underlying cause of death [21]. Since 2005, Finland has followed the WHO guideline limiting the use of pneumonia as an underlying cause of death [14]. Thus, if a person with dementia dies of pneumonia, the underlying cause of death must be registered as dementia [23]. The high prevalence of dementia in LTC and coding differences may partly explain the low proportion of infections listed as cause of death in our study.

Internationally, dementia has been found to be underreported on death certificates [24, 25], even though such reporting has increased in recent decades, and underreporting among nursing home residents is not as common as among those who die in hospital [26]. Autopsies have shown that bronchopneumonia and ischemic heart disease are the most frequent immediate causes of death in people with dementia [11, 27]. Most nursing home residents prefer not to die in hospitals [28]. However, end-of-life hospitalization of nursing home residents is common, and interventions are needed to improve residents’ end-of-life care [29]. We hope that the data provided on mortality and causes of death can help to better predict death and plan timely palliative care in LTC facilities.

One of the strengths of this study was the relatively high number of LTC residents. All residents were assessed by well-trained study nurses. A large number of validated variables were used. The data collection and the methodology used led to high data validity and there was no loss to follow-up. The study sample was representative of LTC residents in terms of age, sex, and cognition status. Furthermore, earlier studies have not compared differences in causes of death in LTC residents between sexes and people with and without dementia.

A limitation of the study was that it took place in only one city and one country, thus potentially limiting the generalizability of the results, even though the sample was representative of LTC residents. All residents were already living in LTC on the study inclusion day, thus, we do not have information on their entry date or length of stay in LTC. The mortality rates could be underestimations due to selection bias because residents with moderate to severe dementia (CDR 2–3), without any proxy available to give informed consent, were excluded from the study. However, due to dementia, most participants needed the consent of a proxy. Another weakness of the study was its reliance on death certificates. Most of the residents in our study did not undergo an autopsy. Certain chronic underlying diseases may have gone undiagnosed due to the poor condition of LTC residents, thus, dementia could be overrepresented. Death certificates written without an autopsy are based on a physician’s assessment of the cause of death. Studies have shown that clinical death certificate information might not be sufficiently reliable [30]. However, because we knew the underlying medical conditions of the residents, and the majority had dementia and CVD, we do not believe that the study’s reliance on death certificates had a significant impact on the results.

## Conclusions and implications

The study confirmed earlier findings indicating that mortality rates for LTC residents are higher, especially for men and residents under 70 years of age than among their community-dwelling counterparts. The all-cause SMR among those with dementia did not differ compared to those without dementia at baseline. The most common cause of death was dementia, followed by CVD. Knowledge about the mortality and causes of death of LTC residents could help improve end-of-life care for residents and help healthcare workers better anticipate death and avoid unnecessary patient transfers and interventions.
